# A Magnetic Resonance Fingerprinting Approach for Simultaneous $T_{1}$‐ and PRFS‐Based 3D MR‐Thermometry

**DOI:** 10.1002/mrm.70392

**Published:** 2026-04-22

**Authors:** Moritz Gutt, Dominik Horstmann, Othmar Belker, Simon Schröer, Daniel Düx, Frank Wacker, Marcel Gutberlet, Bennet Hensen

**Affiliations:** ^1^ Institute of Diagnostic and Interventional Radiology Hannover Medical School Hannover Germany; ^2^ Research Campus STIMULATE Magdeburg Germany

**Keywords:** magnetic resonance fingerprinting, microwave ablation, MR thermometry

## Abstract

**Purpose:**

To evaluate a three dimensional dual echo magnetic resonance fingerprinting sequence for simultaneous proton resonance frequency shift (PRFS) and $T_{1}$ thermometry during microwave ablation with $T_{1}$ improving robustness near gas bubble induced susceptibility changes while PRFS provides high precision elsewhere.

**Methods:**

An ex vivo bovine liver was heated at 40 W for 8 min. A 3D stack‐of‐spiral FLASH‐MRF acquisition on a 3 T system produced simultaneous PRFS‐ and $T_{1}$‐based thermometry. Two fiber optic probes served as references. $T_{1}$ was calibrated to temperature using a parenchymal region in PRFS thermometry. Thermal dose was computed as CEM43 and compared with post‐cooling inversion‐recovery imaging.

**Results:**

The $T_{1}$–temperature slope was 4.81ms/°C. In non‐heated parenchyma, temporal standard deviation was 0.5±0.2°C for PRFS and 1.3±0.2°C for $T_{1}$. At probe 1, RMSE was 0.9°C for PRFS and 2.2°C for $T_{1}$. At a susceptibility‐prone probe, PRFS RMSE was 15.3°C while $T_{1}$ RMSE was 2.1°C regarding only time points up to a CEM43 threshold of 240 min. Dose maps achieved Dice overlap of 67.13% for PRFS and 73.80% for $T_{1}$.

**Conclusion:**

A single three dimensional MRF acquisition provided complementary temperature information. PRFS delivered high precision where phase was stable. $T_{1}$ remained monotonic near susceptibility and improved dose overlap. The framework encoded $B_{1}^{+}$ and enabled in‐session $T_{1}$ calibration from PRFS.

## 1 Introduction

Thermal tumor ablation is an established option for patients with limited metastatic burden or early‐stage hepatocellular carcinoma [1, 2]. To minimize local recurrence and support progression‐free survival, complete ablation with an adequate safety margin (approximately 5 mm) is desirable [3]. Reliable achievement of this goal can be facilitated by MR‐guided real‐time temperature monitoring. While most MR‐related parameters are temperature dependent [4] and might thus be feasible for MR‐thermometry, proton resonance frequency shift (PRFS)–based thermometry is most widely used [5]. PRFS is particularly attractive because the water resonance frequency varies approximately linearly with temperature and is relatively insensitive to many tissue‐specific properties [6].

However, several limitations of PRFS are well recognized. First, because PRFS with temperature in fat is negligibly small [7], no direct temperature readout is obtained in adipose tissue. Second, as a phase‐based measurement, PRFS is vulnerable to magnetic susceptibility‐induced background phase changes (e.g., from gas formation), which can result in non‐physiological apparent cooling or heating [8, 9]. These issues are pronounced during microwave ablation, in which rapid heating promotes vapor formation [10, 11]. Bubble‐related susceptibility artifacts have also been reported during MR‐guided laser interstitial thermal therapy (LITT) [12, 13].

To extend temperature mapping into adipose tissue, simultaneous PRFS and $T_{1}$ estimation has been implemented with single‐reference variable flip‐angle (VFA) sequences, with feasibility shown by Svedin et al. [14] and by Richards et al. [15]. These VFA approaches require a separate $B_{1}^{+}$ map for flip‐angle calibration, which adds time and can introduce misregistration in interventional workflows. The $T_{1}$ estimate relies on magnitude changes relative to a reference image. Magnitude can change with temperature through $M_{0}$, and it can be reduced by $T_{2}^{*}$ shortening from susceptibility variations such as gas bubbles, which together can bias $T_{1}$‐based temperature if not accounted for [16, 17]. $T_{2}^{*}$‐induced bias in single‐reference VFA dynamic $T_{1}$ mapping and correction strategies for this issue have been reported previously by Malmberg et al. [17, 18].

Therefore, a strategy using Magnetic Resonance Fingerprinting (MRF) [19] is proposed. MRF has been developed as an efficient framework for quantitative multi‐parametric mapping. It allows for $B_{1}^{+}$ deviations to be encoded directly into the framework [20]. MRF has been used for thermometry in two‐dimensional settings, including $T_{1}$‐based thermometry near deep‐brain‐stimulation leads [21], and it has been adapted to achieve high‐precision PRFS thermometry [22]. Here, a three‐dimensional stack‐of‐spiral MRF sequence was designed to deliver simultaneous $T_{1}$ and PRFS thermometry in a single acquisition. PRFS provides precision where phase remains reliable, and $T_{1}$ offers robustness near susceptibility changes. To the authors' best knowledge, this constitutes the first three‐dimensional MRF thermometry for ablation monitoring and the first use of simultaneous $T_{1}$ thermometry to address susceptibility artifacts during microwave ablation.

## 2 Theory

### 2.1 PRFS Thermometry Model

PRFS thermometry estimates temperature change (ΔT) from phase change (Δϕ) under the assumption that phase accrual arises from the temperature‐dependent chemical shift of bulk water. For a gradient‐echo acquisition with echo time TE at field strength $B_{0}$,

(1) $$\Delta T\;=\;\frac{\Delta \phi }{\alpha \;\gamma \;B_{0}\;\mathrm{TE}},$$

where γ is the gyromagnetic ratio and α≈−0.01ppm/°C is the water PRF coefficient [4]. In practice, the measured phase contains additional terms,

(2) $$\Delta \phi \;=\;\Delta \phi_{T}\;+\;\Delta \phi_{\chi }\;+\;\Delta \phi_{B_{0}}\;+\;\Delta \phi_{\text{motion}}\;+\;\varepsilon ,$$

with contributions from susceptibility changes ($\Delta \phi_{\chi }$, e.g., gas formation), background field drift ($\Delta \phi_{B_{0}}$), motion ($\Delta \phi_{\text{motion}}$), and noise (ε). Only $\Delta \phi_{T}$ maps linearly to temperature. Long echo times increase temperature sensitivity, with the optimal echo time being $\mathrm{TE}=T_{2}^{*}$ [4].

### 2.2 Susceptibility Changes During Microwave Ablation

During thermal ablation, local heating can generate gas bubbles within the heated tissue. The large susceptibility contrast between gas and tissue perturbs the static magnetic field, which induces phase offsets in gradient‐echo signals used for PRFS thermometry (see Equation 2). The perturbation is inherently nonlocal. Each bubble behaves as a susceptibility inclusion that produces a dipolar field aligned with $B_{0}$, so the resulting phase pattern extends well beyond the voxels that contain gas. Apparent temperature errors can appear several millimeters to centimeters away from the bubble and cause non‐physiological cooling or heating. During clinical microwave ablation, bubbles arise primarily at the active applicator tip, where boiling is initiated and cause temperature underestimation in the direction of $B_{0}$ and overestimation perpendicular to $B_{0}$. Similar bubble‐related susceptibility artifacts also occur during MR‐guided LITT, associated with gas introduced during probe placement or emerging during heating [8, 9, 10, 13, 23].

### 2.3 $T_{1}$‐Based Thermometry Model

Within a moderate temperature range and prior to coagulation, the longitudinal relaxation time exhibits an approximately linear dependence on temperature [4]. A local calibration model is adopted:

(3) $$T_{1}\left(T\right)\;\approx \;T_{1,0}+\beta \;\Delta T$$

where T denotes the absolute temperature, $T_{1,0}$ the baseline $T_{1}$ value and β the slope (ms/°C). The corresponding temperature change estimated from $T_{1}$ is

(4) $$\Delta T_{T1}\;=\;\frac{T_{1}-T_{1,0}}{\beta }.$$

Because β is tissue‐dependent and can deviate from linearity after irreversible damage, calibration should be restricted to a conservative range (which could be chosen based on thermal dose [24]). T1‐based estimates are comparatively insensitive to susceptibility changes [16] but typically exhibit less accuracy than PRFS [25]. This is because susceptibility‐induced $\Delta B_{0}$ directly adds a PRFS phase term, whereas $T_{1}$ is inferred from magnitude evolution and is mainly affected indirectly via $T_{2}^{*}$ loss (SNR degradation) [26].

### 2.4 MRF Signal Model

Magnetic resonance fingerprinting varies sequence parameters (e.g., flip‐angle, inversion) to encode tissue properties into a time series. The complex signal for a voxel with parameters θ is generated by Bloch simulation, yielding an N‐frame time course $s\left(\theta \right)\in {\mathbb{C}}^{N}$ [19].

The parameters necessary for the simulation are dependent on the sequence type and parameters. In case of a Fast Low‐Angle Shot (FLASH) sequence, the signal is mainly dependent on $\theta =\left(M_{0},T_{1},T_{2}^{*},B_{1}^{+}\right)$. When using a fixed echo time, the influences of $M_{0}$ and $T_{2}^{*}$ cannot be separated and correspond to a multiplication of s(θ) with a scalar value [20]. A dictionary $D=\left[s\left(\theta_{1}\right),\ldots ,s\left(\theta_{P}\right)\right]$ is formed and the parameter combination $\hat{\theta }$ that best represents a voxel's tissue is computed with a pattern matching algorithm. Commonly, the entry with the highest inner product between the normalized dictionary entry and the measured signal curve $s_{\text{meas}}$ is chosen:

(5) $$\hat{\theta }=\arg\max_{\theta_{j}}\;\left|\frac{\left\langle s\left(\theta_{j}\right),s_{\text{meas}}\right\rangle }{|s\left(\theta_{j}\right)|}\right|$$

The complex inner product

(6) $$M_{0}^{*}=\frac{\left\langle s\left(\hat{\theta }\right),s_{\text{meas}}\right\rangle }{|s\left(\hat{\theta }\right)|}$$

contains magnitude information dependent on $M_{0}$ and $T_{2}^{*}$ decay and phase information dependent on off‐resonance effects. The phase information can be used for PRFS‐based thermometry as described in Equation (1).

### 2.5 Subspace Representation and Image Reconstruction

Dictionary time courses are highly correlated temporally, so a singular value decomposition (SVD) can be used to obtain a temporal basis $U_{K}\in {\mathbb{R}}^{N\times K}$ that captures the dominant subspace. Compression using this basis can be performed on k‐space data, thus reducing the number of frames needed to be reconstructed [27].

Let y denote the measured multi‐coil k‐space data and E the operator that includes (non‐Cartesian) Fourier transforms, sampling masks, and coil sensitivities. The following linear inverse problem can be formulated to estimate the coefficient images:

(7) $$\hat{c}\;=\;\arg\min_{c}\;∥{\mathrm{EU}}_{K}c-y{∥}_{2}^{2}\;+\;\sum_{i}\lambda_{i}R_{i}\left(c\right),$$

where the last term denotes optional regularization functions $R_{i}$ with weights $\lambda_{i}$ [28].

The reconstructed coefficient images are then matched to the subspace‐compressed dictionary as in Equation (5).

## 3 Methods

### 3.1 Experimental Setup

**FIGURE 1:**
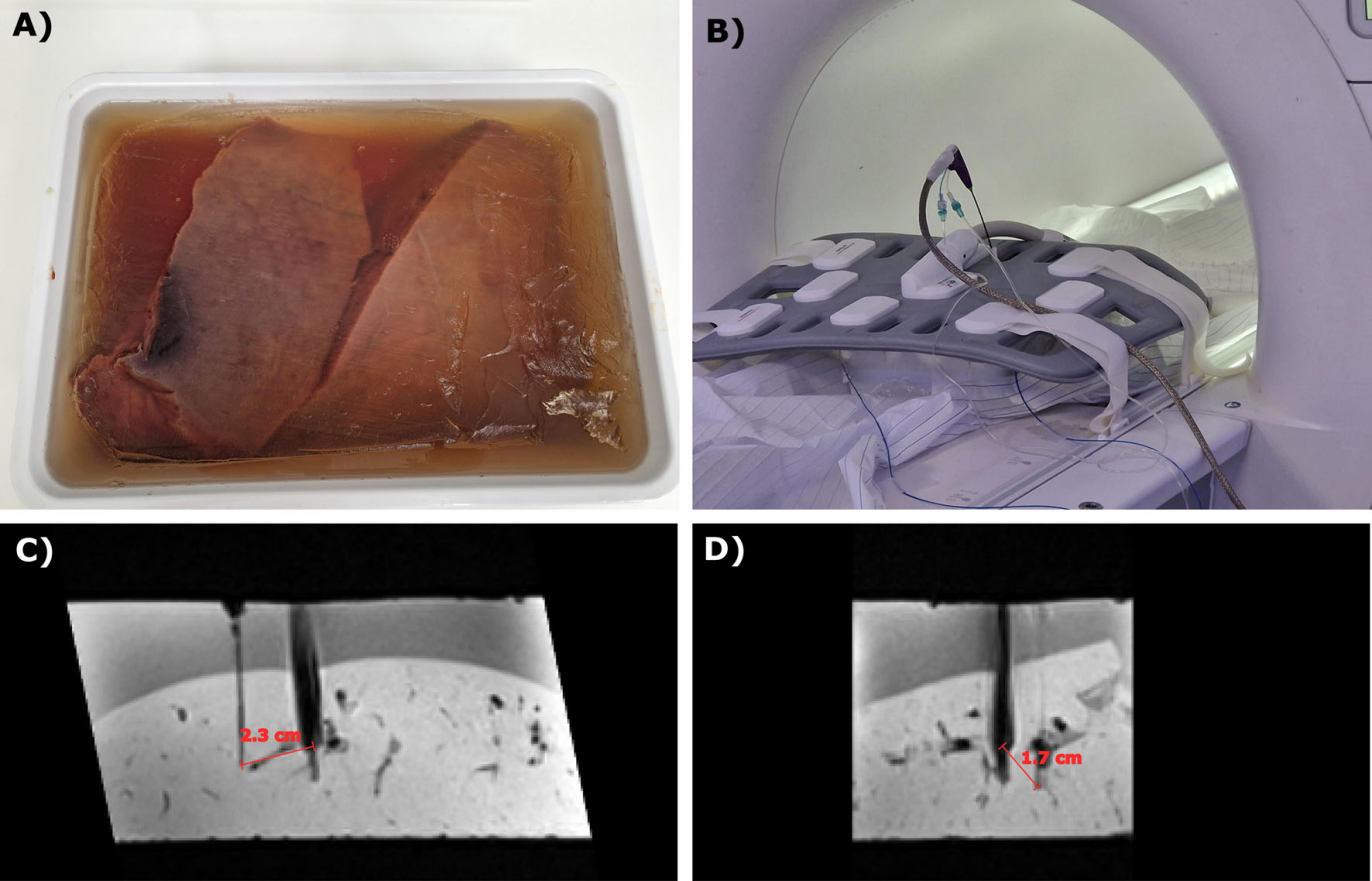
(A) the liver phantom cast in gelatin and (B) the experimental setup with a body matrix placed on top of the phantom and the microwave antenna and the two optical temperature sensors inserted. (C) and (D) show magnitude images of the two temperature sensors in oblique slices and highlight their distance to the active part of the microwave antenna (2.3 and 1.7 cm).

A piece of ex vivo bovine liver was embedded in gelatin to provide mechanical stability. Microwave ablation was performed with a clinically approved microwave generator system (MWG, ECO‐200, ECO Medical Technologies, China) at 40 W for 8 min. To minimize RF interference, the microwave generator was placed outside the scanner room. The 5 m microwave cable was routed through the waveguide and equipped with shielding measures that included ferrite chokes, copper tape, and copper mesh [29]. Two fiber‐optic temperature probes (FOTEMPOEM‐PLUS, 200 μm fiber, 300 μm × 300 μm sensitive tip area, Weidmann Technologies Deutschland GmbH) were positioned approximately 2.3 and 1.7 cm from the active tip to provide independent reference temperatures. Figure 1 shows the experimental setup. Before ablation, a high‐resolution scan was acquired to localize the sensors. After ablation and cooling to room temperature, $T_{1}$ in a representative slice through the ablation zone was estimated by least‐squares fitting of inversion‐recovery (IR) images acquired at multiple inversion times to determine an inversion time that nulls the ablation‐zone signal. Subsequently, a multi‐slice inversion‐recovery acquisition ($1.0\times 1.0\;{\mathrm{mm}}^{2}$ in‐plane resolution, 1.0mm slice thickness and 20% slice gap) of the entire volume was performed with TI=223ms selected based on this fit. The IR volume was then manually segmented to delineate the ablation zone. This approach is supported by prior work showing that coagulation irreversibly alters tissue $T_{1}$, both in vivo and ex vivo [24, 30].

### 3.2 Sequence Design

Imaging was performed on a 3 T clinical scanner (MAGNETOM Vida, Siemens Healthineers, Germany). A 3D stack‐of‐spiral FLASH‐based MRF sequence was designed with a nonselective adiabatic inversion followed by a flip‐angle train of N=128 frames. Repetition time was TR=15ms. Two echoes were acquired per frame with ${\mathrm{TE}}_{1}=0.8\;\mathrm{ms}$ using spiral‐out and ${\mathrm{TE}}_{2}=12.7\;\mathrm{ms}$ using spiral‐in. The field of view was 320×320×40 mm^3^ with 2.5 mm isotropic resolution. Undersampling was $R_{z}=2$ through plane and $R_{\text{in}‐\text{plane}}=14$. The acquisition time per thermometry time point can be calculated as $\left(N\cdot \mathrm{TR}+T_{\mathrm{inv}}\right)\cdot \left(N_{z}/R_{z}\right)=\left(128\times 15\;\mathrm{ms}+21.25\;\mathrm{ms}\right)\times \left(16/2\right)=15.53\;s$, where $N_{z}$ denotes the number of partitions and $T_{\mathrm{inv}}$ the time added by the inversion pulse once per flip‐angle train. The base spiral was designed with a maximum frequency constraint of 2 kHz using the method of Pipe et al. [31] and was rotated by the golden angle (137.5°) between subsequent acquisitions. The flip‐angle schedule was constrained to 5° to 40° and was optimized regarding the Cramér–Rao lower bound [32] for combinations of $T_{1}\in \left[400:100:800\right]$ ms (values from 400 to 800 ms with a step size of 100 ms) and $B_{1}^{+}\in \left[0.75:0.25:1.25\right]$. Sequence optimization was implemented in Python using the CasADi toolbox [33] and was based on MATLAB code provided by van Riel et al. [20]. The flip‐angle train was executed once before data collection to approach steady state. Five minutes of baseline data were acquired before and additional 5 min of cooling were acquired after ablation. Figures S1 and S2 show the optimized flip‐angles as well as a graph representation of the reconstruction pipeline.

### 3.3 Image Reconstruction and Parameter Estimation

After the experiment, raw data was exported from the scanner and image reconstruction was performed offline. A dictionary was simulated with the sequence timings, covering $T_{1}\in \left[200:2:1200\right]$ ms and $B_{1}^{+}\in \left[0.2:0.02:2.0\right]$. Dictionary time courses were compressed by singular value decomposition and the leading K=5 singular vectors were retained which captured more than 99.9% of the dictionary energy. Coefficient images were estimated by solving the subspace reconstruction in Equation (7) using the BART toolbox [34]. Because undersampling is a major source of error in MRF [35], the first ten time points were reconstructed together as one image to provide a baseline with relatively low undersampling. Each subsequent reconstruction was warm started with the previous time point. An $\ell_{1}$ wavelet penalty and a locally low‐rank penalty were applied in the spacial dimensions. The use of $\ell_{1}$ wavelet and locally low‐rank regularization together with warm starting was previously proposed by Lima da Cruz et al. [36] to reduce undersampling artifacts. The subspace‐compressed voxel time courses were matched to the compressed dictionary by the normalized inner product in Equation (5).

### 3.4 Temperature Mapping

PRFS temperature maps were calculated according to Equation (1) from the complex inner‐product results (Equation 6) for each echo and were combined using the method of Madore et al. [37]. Phase drift correction was performed by subtracting the calculated temperature change in a 8×8×8 non‐heated ROI. Temporal phase unwrapping was performed relative to the baseline time point. A 4×4×4 voxel region in parenchyma was selected for calibration of $T_{1}$ to temperature change. The region was chosen manually such that heating occurred but it was visually not affected by susceptibility‐related artifacts in the PRFS temperature maps. To ensure that calibration is not influenced by tissue coagulation, only data points with CEM43 [38] threshold below 240 min (based on PRFS temperature maps) entered a linear regression of $T_{1}$ versus PRFS temperature to estimate $T_{1,0}$ and β from Equation (3). The calibrated $T_{1}$ based temperature was then computed for all voxels using Equation (4). Calibration parameters were computed once for the experiment and applied to the full time series. For comparison with the fiber‐optic sensors, the directly neighboring voxel that best matched (lowest RMSE) $T_{1}$ and PRFS was chosen and kept fixed for the whole time [39]. Temperature accuracy was evaluated below CEM43 threshold of 240 min (based on sensor readings). Additionally, the mean standard deviation in non‐heated tissue was assessed using the same ROI as for the phase drift correction.

### 3.5 Thermal Dose and Ground Truth

Per‐voxel temperature histories from PRFS and from $T_{1}$ were converted to CEM43. Binary dose masks were defined by a threshold of 240 min. The post‐cooling IR images were manually segmented and interpolated to the thermometry images. The IR ablation zone served as ground truth for overlap analyses.

## 4 Results

### 4.1 Calibration of $T_{1}$ to Temperature

**FIGURE 2:**
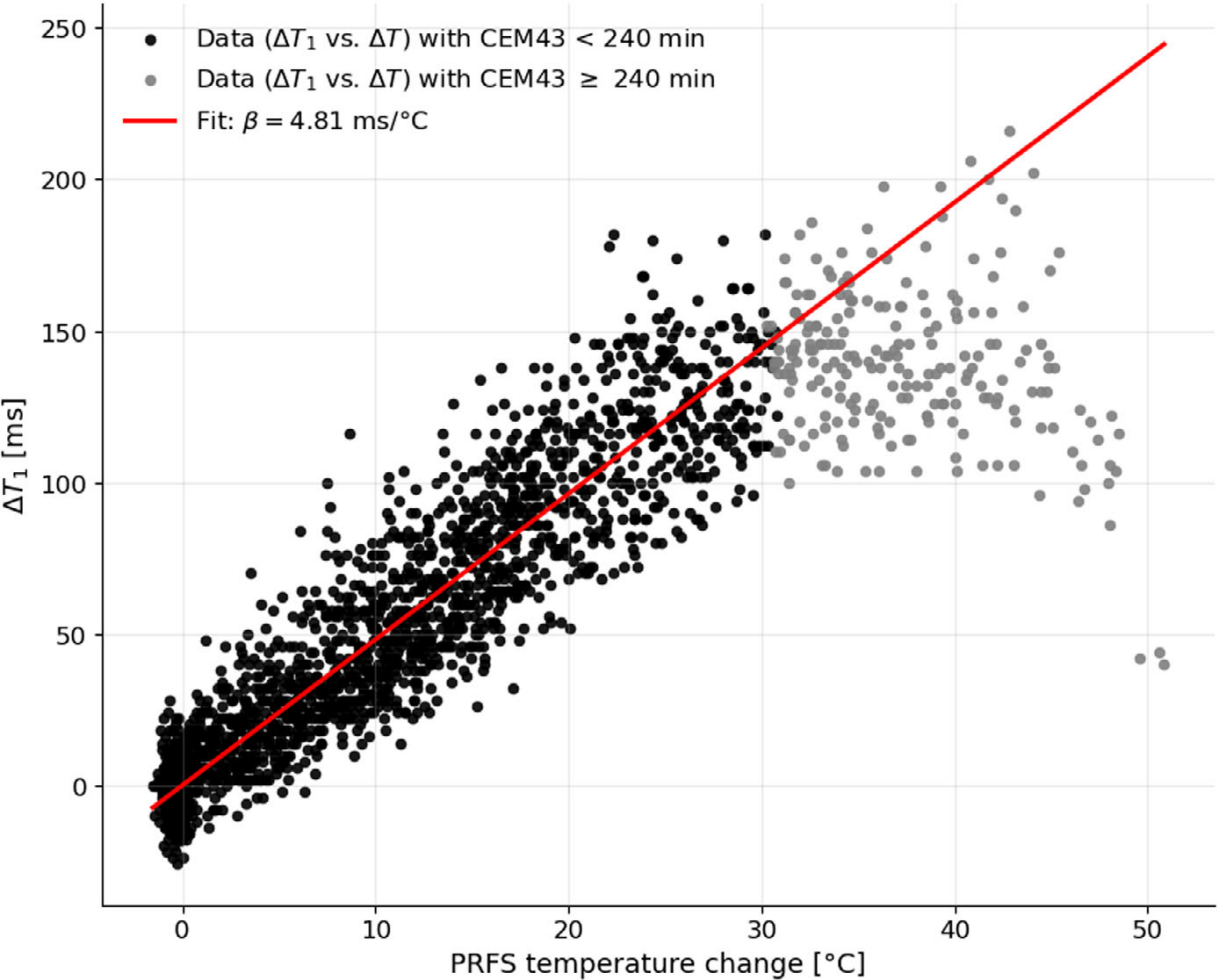
Scatter plot between $T_{1}$ and PRFS‐derived temperature changes in the calibration region. The red line visualizes the linear fit from data points below CEM43 of 240 min (black points). Non‐linear effects can be seen at higher temperatures due to tissue coagulation.

Within the calibration region, $T_{1}$ increased linearly with PRFS‐derived temperature below 43°C. The fitted slope was $\beta =4.81\;\mathrm{ms}{}^{\circ}C^{-1}$ which corresponds to $1.00\;\%\;{}^{\circ}C^{-1}$ with $R^{2}=0.88$ (Figure 2).

### 4.2 Behavior in Susceptibility‐Prone Regions

**FIGURE 3:**
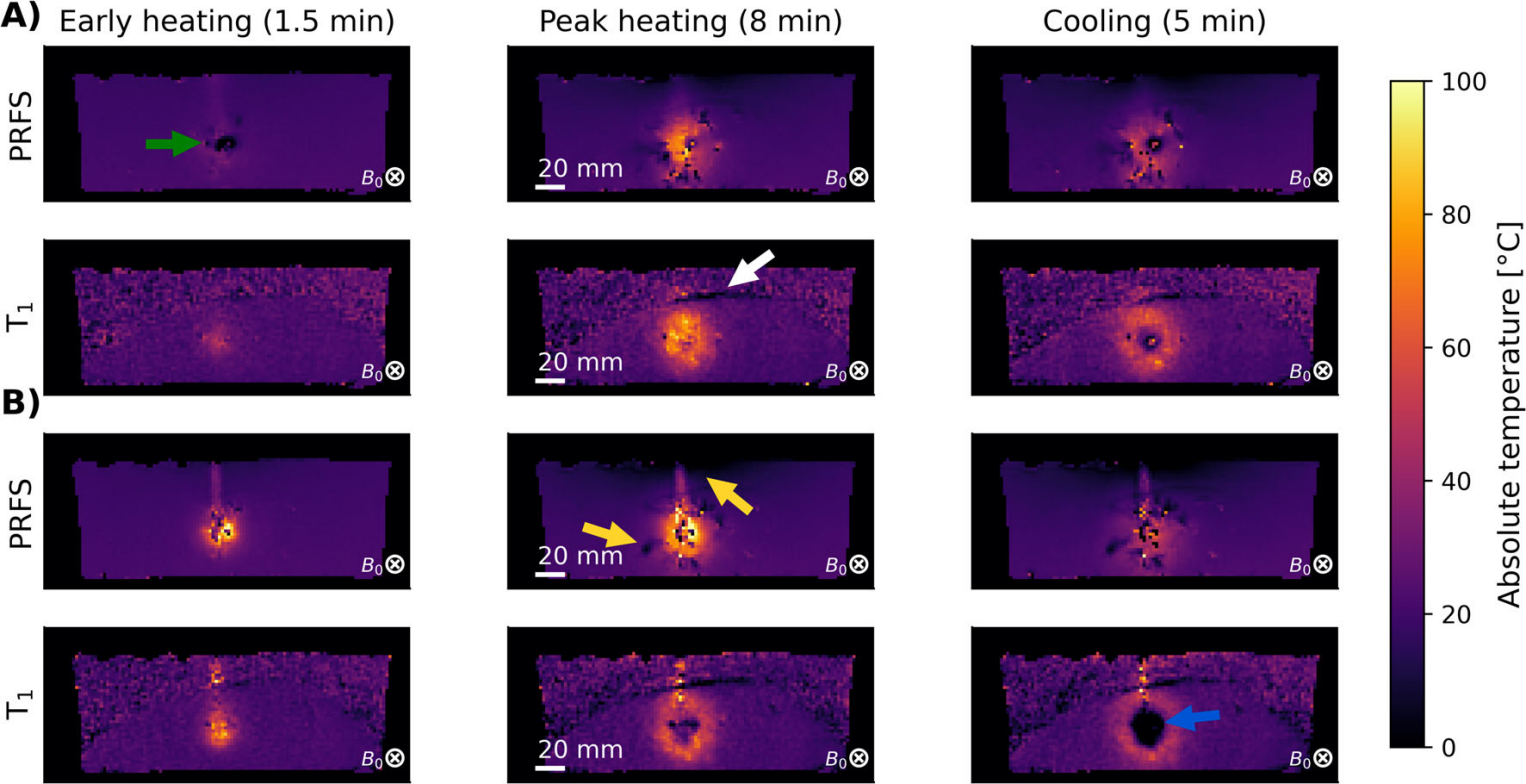
Magnetic susceptibility effects during microwave ablation in (A) an outer slice (1 cm from the needle tip) and (B) the central slice with the needle tip inside. Rows show PRFS‐based and $T_{1}$‐derived temperature maps at 1.5 and 8 min after ablation onset and after 5 min of cooling. Early in heating, gas bubbles form at the needle tip and cause apparent cooling in slices farther from the applicator (green arrow). With continued heating, bubbles propagate along empty vessels and the needle tract, producing strong susceptibility changes (yellow arrows). $T_{1}$‐based temperature maps display apparent central cooling within the ablation zone (blue arrow) consistent with non‐linear $T_{1}$ versus temperature changes occurring after tissue coagulation. The liver phantom expanded slightly due to heating which causes the dark stripe at the edge between liver and gelatin (white arrow). The $B_{0}$ field is perpendicular to the shown slices.

Example time points demonstrated pronounced magnetic susceptibility effects in PRFS‐derived temperature maps, particularly near larger vessels. The corresponding $T_{1}$‐based temperature maps remained smooth and monotonic in the same regions. A visual comparison is shown in Figure 3.

### 4.3 Temperature Accuracy

In non‐heated parenchyma, the mean temporal standard deviation of PRFS‐derived temperature was 0.5±0.2°C. The corresponding value for the $T_{1}$‐based temperature was 1.3±0.2°C.

**FIGURE 4:**
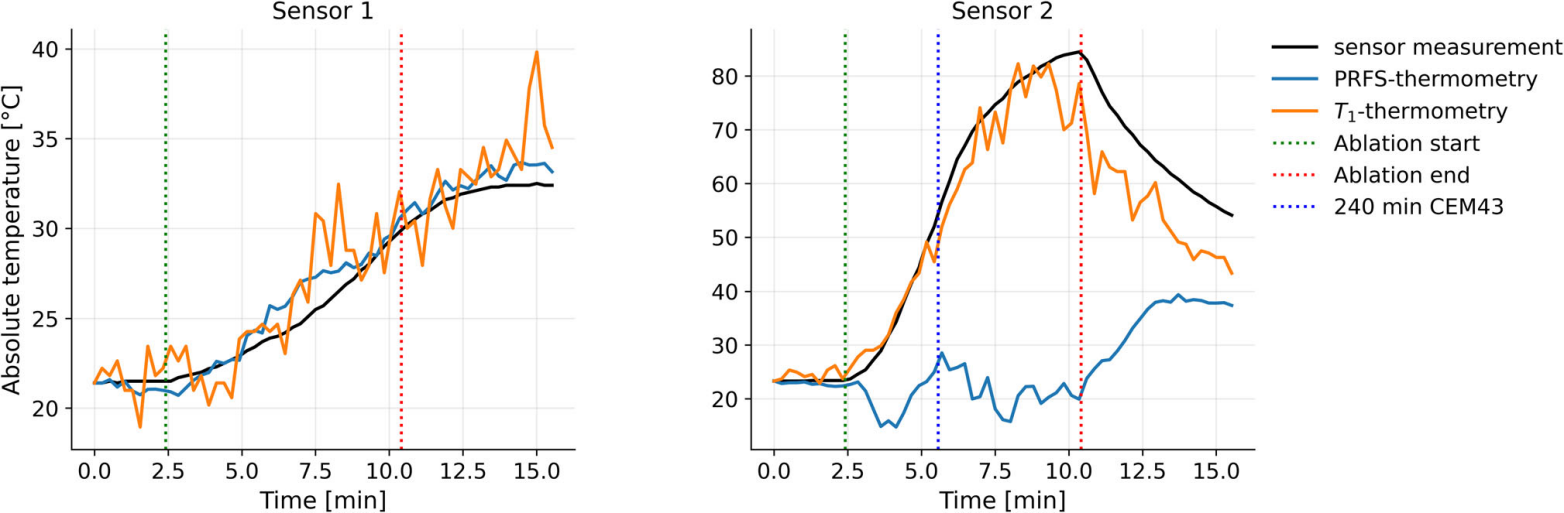
Temperature time courses from fiber‐optic probes compared with PRFS‐ and $T_{1}$‐based estimates. Sensor 1 (left) was positioned 2.3 cm from the ablation center and Sensor 2 (right) 1.7 cm. At Sensor 2, PRFS thermometry was strongly affected by susceptibility changes that emerged shortly after ablation started, yielding non‐physiological apparent cooling. The PRFS curve increased again after power delivery ended. $T_{1}$ becomes unreliable at higher temperatures and deviates from the sensor readings due to tissue coagulation. The blue dotted vertical line marks the time when CEM43 of 240 min is reached according to the sensor readings. Note that only approximately 2.5 of the 5 min without heating are shown here as the first ten time points were used to reconstruct the baseline image.

Figure 4 shows the measured temperature curves from the fiber‐optic probes compared to the PRFS‐ and $T_{1}$‐based thermometries. At the first fiber‐optic probe location, PRFS agreed with the reference with a root‐mean‐square error (RMSE) of 0.9°C while the $T_{1}$‐based temperatures showed an RMSE of 2.2°C. The second probe was located in a susceptibility‐prone region. Considering all time points up to the moment when the local CEM43 reached 240 min, the RMSE was 15.3°C for PRFS and 2.1°C for $T_{1}$‐based thermometry. When additionally including time points beyond 240 min, the RMSE increased to 35.8°C for PRFS and 7.5°C for $T_{1}$. Temperature maps in the slices with the probes can be seen in Supporting Figure S3.

### 4.4 Ablation‐Zone Prediction From Thermal Dose

**FIGURE 5:**
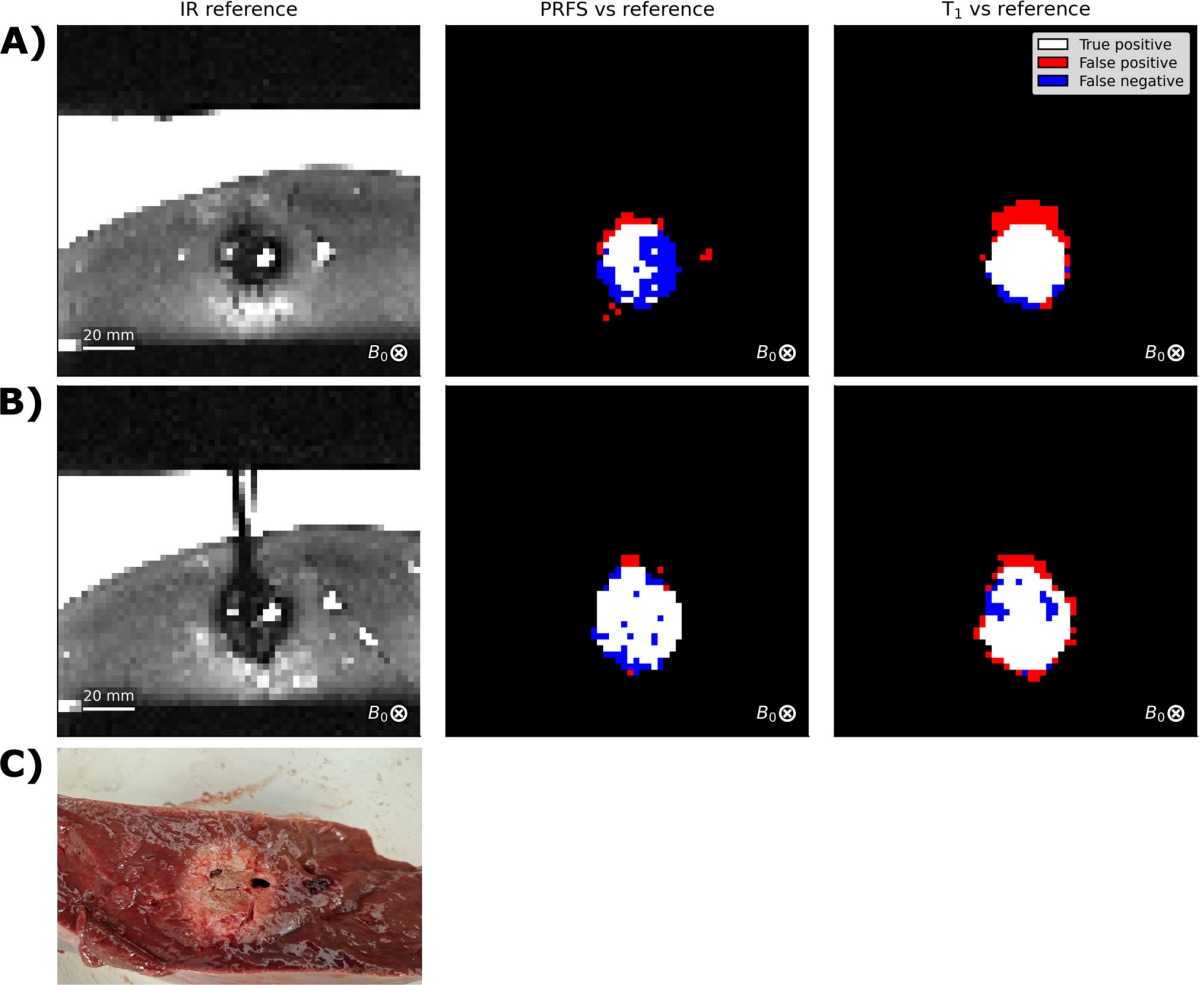
Ablation zone comparison against IR reference in the same slices as in Figure 3 with (A) being 1 cm from the needle tip and (B) being the central slice with the needle tip inside. Column 1 shows the post‐cooling inversion‐recovery magnitude for anatomic context (down‐scaled to the thermometry resolution). Columns 2 and 3 show voxelwise agreement between CEM43‐based ablation masks and the ground truth segmentation with true positives in white, false positives in red and false negatives in blue. The influence of susceptibility effects can be seen especially in the PRFS ablation zone of the outer slice. The $B_{0}$ field is perpendicular to the shown slices. (C) shows an image of an axial cut through the center of the ablation zone.

Dose maps computed from PRFS temperatures yielded a Dice coefficient of 67.13%. Dose maps computed from $T_{1}$‐based temperatures yielded a Dice coefficient of 73.80% (Figure 5).

## 5 Discussion

This study showed that a single three‐dimensional MRF acquisition delivered complementary PRFS‐ and $T_{1}$‐based temperature information during microwave ablation. In regions without visible susceptibility, PRFS provided lower noise and smaller error at the probe. In susceptibility‐affected regions near vessels, PRFS exhibited non‐physiological excursions, whereas the $T_{1}$‐based estimate remained monotonic within the calibrated range. After conversion to thermal dose, the stable $T_{1}$ trend improved overlap with the post‐cooling inversion‐recovery reference.

The observed behavior is consistent with the underlying physics. PRFS measures phase and offers high temperature precision when the background phase is stable. $T_{1}$ reflects magnitude evolution and is less sensitive to local susceptibility, though it shows higher variance for a given acquisition time. The measured slope of approximately $1\;\%\;{}^{\circ}C^{-1}$ supported a simple linear conversion within the non‐coagulative range and enabled consistent temperature tracking where PRFS failed. At higher temperatures the linear relationship between $T_{1}$ and temperature is not necessarily given anymore due to tissue coagulation (see Figures 2 and 4). This can lead to an underestimation of the ablation extent if heating is rapid, because the coagulation‐induced $T_{1}$ decrease can counteract the temperature‐driven $T_{1}$ increase before it is fully captured by the $T_{1}$‐based thermometry. A small bias in the $T_{1}$ estimates may also arise because $T_{1}$ can change during acquisition of a 3D thermometry time point, whereas the dictionary assumes constant $T_{1}$ within each volume update.

Although experiments were performed at 3 T, translation to 1.5 T should be feasible, but PRFS sensitivity scales with $B_{0}$ and would therefore be lower [4]. Flip‐angle optimization should also be adapted because baseline $T_{1}$ depends on field strength.

Two practical features are relevant for interventions. First, transmit‐field inhomogeneity can be handled within the MRF model. $B_{1}^{+}$ scaling is encoded in the dictionary and estimated during matching, which removes a separate mapping step that can misregister in the presence of motion or hardware manipulation. Second, simultaneous acquisition of PRFS and $T_{1}$ enables on‐the‐fly calibration of $T_{1}$ against PRFS within the same session and geometry. This reduces dependence on external calibration data and ties the conversion to the current heating conditions.

Susceptibility behavior in the ex vivo model warrants consideration. Empty vessels can conduct steam, producing extended phase disturbances and large apparent cooling or heating in PRFS maps. In clinical microwave ablation, gas formation is expected to concentrate at the active part of the applicator and to create a more localized dipole artifact. The ex vivo setting therefore likely overstates the spatial spread of artifacts. The advantage of $T_{1}$ near susceptibility should remain relevant in vivo, although the extent of the affected region may be smaller.

Several additional limitations should be noted. Experiments were performed ex vivo without perfusion or motion, and the sample size was limited. Reconstruction was performed offline rather than in real time, which precluded intraprocedural use. Dictionary generation and SVD compression required 1 min. Offline reconstruction required 40 s per thermometry time point, with an additional 14 s for dictionary matching. All computations were performed on a workstation equipped with an NVIDIA RTX A6000 GPU.

Future work should focus on fusion rather than parallel reporting. One practical approach is to estimate the susceptibility dipole from the difference between PRFS‐ and $T_{1}$‐based temperature maps, followed by correction with the algorithm of Dantas et al. [9] to obtain artifact‐reduced PRFS maps. Prospective validation in perfused in vivo liver with motion management is also needed.

Taken together, the results indicate that simultaneous PRFS and $T_{1}$ thermometry within an MRF framework can improve ablation assessment near susceptibility while preserving PRFS precision elsewhere. This supports development of artifact‐aware thermometry that exploits the strengths of both signals in a single acquisition.

## 6 Conclusion

A single three‐dimensional MRF acquisition enabled simultaneous PRFS‐ and $T_{1}$‐based thermometry during microwave ablation. PRFS provided high precision where background phase was stable, while the $T_{1}$‐based estimate remained monotonic and usable in susceptibility‐prone regions. The combination improved dose‐based ablation‐zone prediction relative to PRFS alone and supported agreement with post‐cooling inversion‐recovery imaging. Practical advantages included direct handling of $B_{1}^{+}$ within the MRF framework and the ability to calibrate $T_{1}$ to temperature from PRFS in the same geometry. Limitations included the ex vivo setting and offline reconstruction that precluded intraprocedural use. Future work should target real‐time implementation and artifact‐aware fusion that uses $T_{1}$ to stabilize PRFS in regions with susceptibility, with validation in perfused in vivo liver.

## Funding

This work was supported by the Federal Ministry of Education and Research within the research campus STIMULATE under the grant number 13GW0473B.

## Conflicts of Interest

The authors declare no conflicts of interest.

## Supporting information


**Figure S1:** Optimized flip‐angle train. The visualization is similar to the one used by van Riel et al. where the arrows show the main $T_{1}$‐encoding part (red arrow), the main $B_{1}^{+}$‐encoding part (green arrow) and the main part for the magnetization to recover (light blue arrow).
**Figure S2:** Schematics of the reconstruction pipeline.
**Figure S3:** Temperature maps in the slices that contain (A) sensor 1 and (B) sensor 2 at 1.5 min and 8 min after starting the ablation as well as after 5 min of cooling. Sensor positions are marked with an asterisk.

## Data Availability

The data that support the findings of this study are available from the corresponding author upon reasonable request.
