# Supplementary material for: A Magnetic Resonance Fingerprinting Approach for Simultaneous T1‐ and PRFS‐Based 3D MR‐Thermometry
Source: Magn Reson Med. 2026 Apr 22;96(3):1274–82. doi: 10.1002/mrm.70392 (PMC13327483; doi:10.1002/mrm.70392)
Supplement: Supplementary file 1 — Figure S1: Optimized flip‐angle train. The visualization is similar to the one used by van Riel et al. where the arrows show the main T1‐encoding part (red arrow), the main B1+‐encoding part (green arrow) and the main part for the magnetization to recover (light blue arrow). Figure S2: Schematics of the reconstruction pipeline. Figure S3: Temperature maps in the slices that contain (A) sensor 1 and (B) sensor 2 at 1.5 min and 8 min after starting the ablation as well as after 5 min of cooling. Sensor positions are marked with an asterisk. [file MRM-96-1274-s001.pdf]

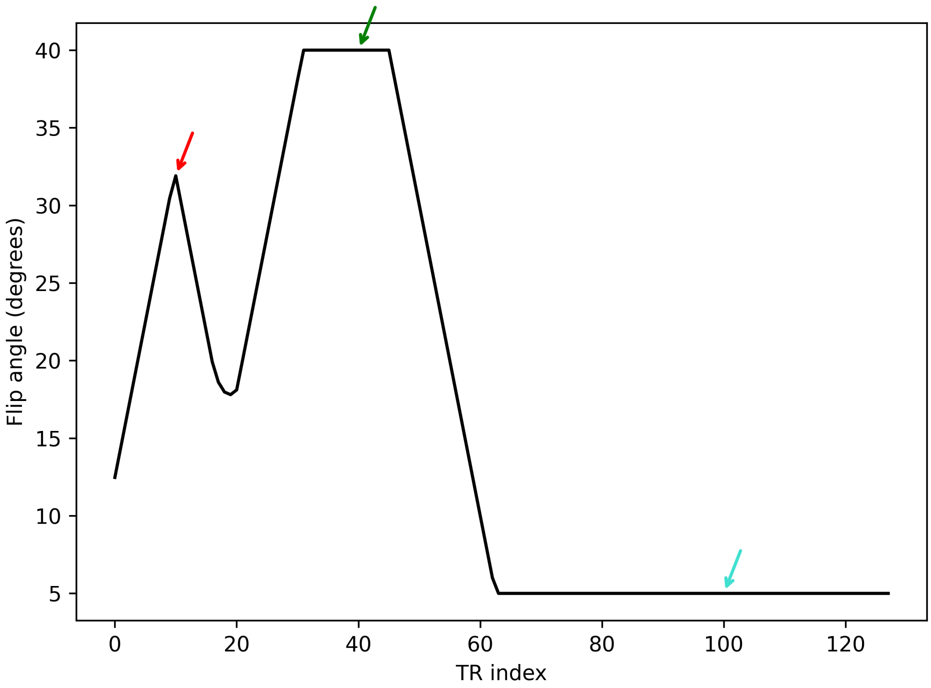

Figure S1. Optimized flip-angle train. The visualization is similar to the one used by van Riel et al. where the arrows show the main T1-encoding part (red arrow), the main B+1-encoding part (green arrow) and the main part for the magnetization to recover (light blue arrow).

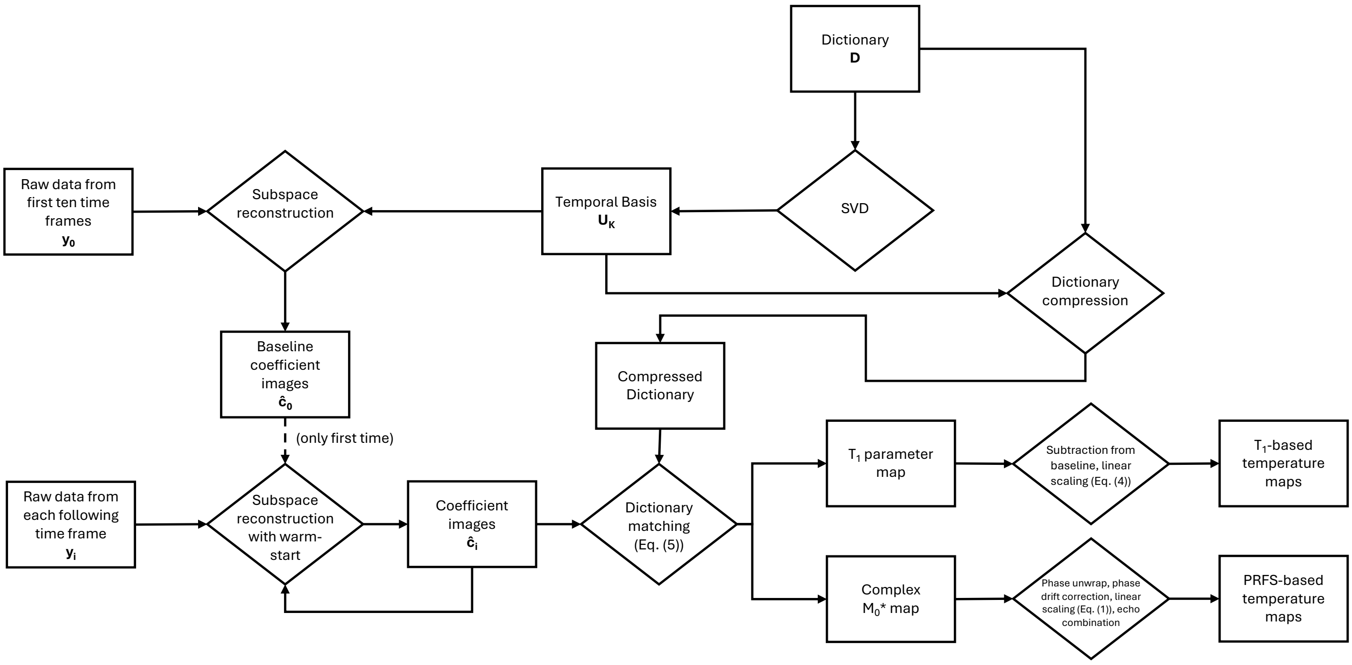

Figure S2. Schematics of the reconstruction pipeline.

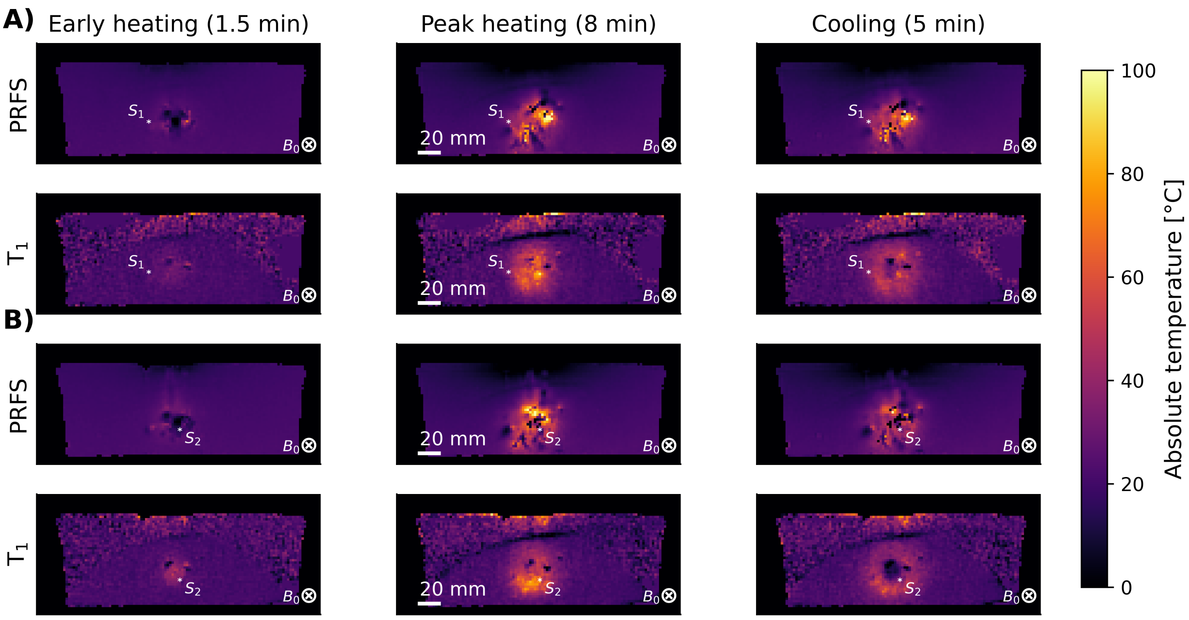

Figure S3. Temperature maps in the slices that contain A) sensor 1 and B) sensor 2 at 1.5 min and 8 min after starting the ablation as well as after 5 min of cooling. Sensor positions are marked with an asterisk.
